# A Comparison of MRI Quantitative Susceptibility Mapping and TRUST-Based Measures of Brain Venous Oxygen Saturation in Sickle Cell Anaemia

**DOI:** 10.3389/fphys.2022.913443

**Published:** 2022-08-29

**Authors:** Russell Murdoch, Hanne Stotesbury, Patrick W. Hales, Jamie M. Kawadler, Melanie Kölbel, Christopher A. Clark, Fenella J. Kirkham, Karin Shmueli

**Affiliations:** ^1^ Department of Medical Physics and Biomedical Engineering, University College London, London, United Kingdom; ^2^ Developmental Neurosciences, UCL Great Ormond Street Institute of Child Health, London, United Kingdom; ^3^ Clinical and Experimental Sciences, University of Southampton, Southampton, United Kingdom

**Keywords:** sickle cell anaemia, T2-relaxation-under-spin-tagging, quantitative susceptibility mapping, venous oxygen saturation, validation, magnetic resonance imaging

## Abstract

In recent years, interest has grown in the potential for magnetic resonance imaging (MRI) measures of venous oxygen saturation (Y_v_) to improve neurological risk prediction. T_2_-relaxation-under-spin-tagging (TRUST) is an MRI technique which has revealed changes in Y_v_ in patients with sickle cell anemia (SCA). However, prior studies comparing Y_v_ in patients with SCA relative to healthy controls have reported opposing results depending on whether the calibration model, developed to convert blood T_2_ to Y_v_, is based on healthy human hemoglobin (HbA), bovine hemoglobin (HbBV) or sickle hemoglobin (HbS). MRI Quantitative Susceptibility Mapping (QSM) is an alternative technique that may hold promise for estimating Y_v_ in SCA as blood magnetic susceptibility is linearly dependent upon Y_v_, and no significant difference has been found between the magnetic susceptibility of HbA and HbS. Therefore, the aim of this study was to compare estimates of Y_v_ using QSM and TRUST with five published calibration models in healthy controls and patients with SCA. 17 patients with SCA and 13 healthy controls underwent MRI. Susceptibility maps were calculated from a multi-parametric mapping acquisition and Y_v_ was calculated from the mean susceptibility in a region of interest in the superior sagittal sinus. TRUST estimates of T_2,_ within a similar but much smaller region, were converted to Y_v_ using five different calibration models. Correlation and Bland-Altman analyses were performed to compare estimates of Y_v_ between TRUST and QSM methods. For each method, t-tests were also used to explore group-wise differences between patients with SCA and healthy controls. In healthy controls, significant correlations were observed between QSM and TRUST measures of Y_v,_ while in SCA, there were no such correlations. The magnitude and direction of group-wise differences in Y_v_ varied with method. The TRUST-HbBV and QSM methods suggested decreased Y_v_ in SCA relative to healthy controls, while the TRUST-HbS (*p* < 0.01) and TRUST-HbA models suggested increased Y_v_ in SCA as in previous studies. Further validation of all MRI measures of Y_v_, relative to ground truth measures such as O^15^ PET and jugular vein catheterization, is required in SCA before QSM or TRUST methods can be considered for neurological risk prediction.

## 1 Introduction

Sickle cell anemia (SCA) is an inherited blood disorder characterized by hemolytic anemia, vasculopathy, cognitive difficulties and high incidence of ischemic stroke, and silent cerebral infarction (Debaun and Kirkham 2016; Stotesbury et al., 2019). The mechanism for these complications in SCA are poorly understood and current methods of risk prediction in SCA are non-specific and have not been validated in adults (Stotesbury et.al., 2018; Stotesbury et al., 2021).

Interest has grown in the potential for magnetic resonance imaging (MRI) estimates of venous oxygen saturation (Y_v_), and the closely related oxygen extraction fraction (OEF), to improve neurological risk prediction in SCA (Jordan and DeBaun 2018). Measuring Y_v_ *in-vivo* is challenging, and all oxygen-sensitive MRI techniques rely on models to convert the MRI measures into Y_v_. Questions remain around the validity of these models, particularly in conditions such as SCA where alterations in blood rheology and oxygen-carrying capacity may challenge model assumptions.

In this study we aimed to compare measures of Y_v_ in SCA, and healthy controls (HC) subjects, derived from two MRI methods: T_2_-relaxation-under-spin-tagging (TRUST) and the more recently developed quantitative susceptibility mapping (QSM).

TRUST is based on the principle that the transverse relaxation time of blood (T_2b_) is dependent on its oxygen saturation (Lu et al., 2012) and has been widely used to estimate Y_v._ TRUST applies similar principles to arterial spin labelling (ASL) to isolate the signal from venous blood, and uses T_2_ preparation pulses to acquire signal at a series of effective echo times (eTE). The venous blood T_2_ is then estimated from an exponential fit of the signal from venous blood ( ΔS ) as a function of eTE:

$$\Delta S=\;\Delta S_{0}\cdot e^{eTE\cdot \left(\frac{1}{T_{1b}}-\frac{1\;}{T_{2b}}\;\right)}$$ (1)

Where $\Delta S_{0}$ is the signal intensity difference at eTE = 0 and T_1b_ is the longitudinal relaxation time of venous blood.

Five calibration models have been previously developed to convert the venous blood T_2_ measured using TRUST to Y_v_: one based on bovine-hemoglobin (HbBV) (Lu et al., 2012; Jordan et al., 2016), one on hemoglobin-A (HbA) (Bush et al., 2017), and three derived from hemoglobin-S: HbS_Bush_ (Bush et al., 2018), HbS_Li_ (Li et al., 2020) and HbS_Li-Bush_ (Bush et al., 2021). These models are described in detail in the Supplementary materials. Using TRUST, Y_v_ can appear either increased or decreased in SCA relative to healthy controls depending on the calibration model used (Lin et al., 2022). An overview of published studies applying TRUST in SCA subjects is shown in Table 1.

**TABLE 1:** Overview of the previous literature investigating the effect of sickle cell anemia (SCA) on venous oxygen saturation (Y_v_) and oxygen extraction fraction (OEF) relative to healthy controls (HC). Abbreviations: T2-relaxation-under-spin-tagging (TRUST), asymmetric spin echo (ASE), positron emission tomography (PET).

MRI TRUST Studies					
Author	Year	Calibration model	Number of SCA subjects (age mean ± S.D., years)	Number of healthy control (HC) subjects (age mean ± S.D., years)	Results
Jordan et al. (2016)	2016	HbBV	27 (27.7 ± 5.0)	11 (26.9 ± 5.1)	Y_v_ decreased in SCA vs. HC (52.0 ± 7.5% vs. 63.2 ± 6.1%, *p* < 0.05)
Bush et al. (2018)	2017	HbS_Bush_	33 (21.8 ± 9.0)	37 (27.2 ± 10.6)	Y_v_ increased in SCA vs. HC (73 ± 5% vs. 61 ± 6%, *p* < 0.0001)
Li et al. (2020)	2019	Subject Specific	11 (25 ± 7)	12 (35 ± 7)	No significant OEF differences between SCA and HC (37.5 ± 5% vs. 38.9 ± 5% *p* = 0.31)
Vu et al. (2021)	2021	HbS_Li-Bush_	47 (21.7 ± 7.1)	44 (26.4 ± 10.6)	OEF significantly decreased in SCA vs. HC (27.4 ± 4.1% vs. 36.7 ± 6.0%, *p* < 0.01)
**MRI QSM Studies**					
**Author**	**Year**	**ROI**	**Number of SCA subjects**	**Number of Healthy Control (HC) subjects**	**Results**
Shen et al. (2019)	2019	Straight Sinus	16 (24 ± 7)	11 (25 ± 9)	Y_v_ decreased in SCA vs. HC (69.3% vs. 73.9%, *p* < 0.05)
Murdoch et al. (2021)	2021	Superior Sagittal Sinus	88 (18.4 ± 10.0)	30 (18.0 ± 9.6)	Y_v_ decreased in SCA vs. HC (72.3 vs. 74.5, *p* < 0.005)
**MRI and PET Whole Brain Studies**					
**Author**	**Year**	**Method**	**Number of SCA subjects**	**Number of Healthy Control (HC) subjects**	**Results**
Fields et al. (2018)	2017	ASE	36 (10.0)	20 (11.0)	Whole-brain OEF increased in SCA (42.7 vs. 28.8 *p* < 0.001)
Herold et al. (1986)	1986	O^15^ PET	6 (27.5 ± 8.7)	14 (34.3 ± 7.0)	No significant OEF differences (42 ± 4% vs. 44 ± 7%)

QSM is a more recent MRI technique that reconstructs the spatial distribution of magnetic susceptibility (*χ*) from gradient-echo (GRE) phase images (Shmueli 2020). QSM measures of Y_v_ are based on the observation that hemoglobin in its oxygenated form is weakly diamagnetic, while deoxygenated hemoglobin is strongly paramagnetic. Therefore, the measured *χ* of venous blood relative to the surrounding tissue ( $\Delta \chi_{vein-water}$ _,_ assuming that surrounding tissue has the same *χ* as water), should be directly proportional to the concentration of deoxygenated hemoglobin (Jain et al., 2012). $\Delta \chi_{vein-water\;}$ and Y_v_ are linearly related by the following expression (Weisskoff and Kiihne 1992):

$$Y_{v}=\;1-\;\frac{\Delta \chi_{vein-water}\;-\;\Delta \chi_{oxy-water\;}\cdot Hct\;}{\Delta \chi_{do}\;\cdot \;Hct}$$ (2)

Where hematocrit (Hct) is the percentage of erythrocytes in blood, $\Delta \chi_{do}$ is the *χ* shift between fully oxygenated and de-oxygenated erythrocytes [0.27 × 4π ppm (SI)] and $\Delta \chi_{oxy-water\;}$ is the *χ* shift between oxygenated erythrocytes and water [−0.03 × 4π ppm (SI)] (Weisskoff and Kiihne 1992).

QSM has been shown to be sensitive to changes in Y_v_/OEF in conditions which affect cerebral oxygen metabolism and/or blood flow. Oxygen extraction fraction is defined as the arteriovenous difference in oxygen saturation, and increased OEF corresponds to decreased Y_v_, for a given arterial oxygen saturation. QSM was sensitive to changes in hemispheric venous oxygen saturation in patients with acute stroke: increased QSM-based OEF was observed in cerebral veins in the affected hemisphere relative to the contralateral hemisphere (29.3 ± 3.4% versus 25.5 ± 3.1% *p* < 0.032) (Fan et al., 2020). QSM has also been applied in patients with arteriovenous malformations (AVMs), showing that veins draining AVMs had significantly higher Y_v_ compared to healthy veins as a result of arteriovenous shunting (Biondetti et al., 2019).

Previous work in a pilot study of 6 SCA patients and 6 healthy controls has demonstrated no significant *χ* difference between deoxyhemoglobin in sickle and normal erythrocytes, suggesting that QSM is valid in both SCA and healthy controls (HC) (Eldeniz et al., 2021). To date, only two studies of Y_v_ in SCA have been carried out using QSM, with both studies reporting decreased Y_v_ in SCA relative to HC (Shen et al., 2019; Murdoch et al., 2021).

TRUST is a global measure of Y_v_, derived from T_2_ values fitted within a small region of interest (ROI) in the superior sagittal sinus (SSS) within a single slice. QSM can also be used to derive a similar global measure of Y_v_ by estimating the mean magnetic susceptibility within a ROI in the SSS. In QSM, the ROI can be a three-dimensional volume spanning several slices and including many more voxels than the region used in TRUST.

Aiming to improve our understanding of Y_v_ estimation in SCA, we compared measures of global Y_v_ derived from QSM and TRUST using each of the five calibration models.

## 2 Materials and Methods

### 2.1 Patients

Patients with sickle cell anemia (SCA; hemoglobin-SS) and age matched healthy controls were recruited to two concurrent studies with overlapping MRI protocols between 2016 and 2019: the Sleep Asthma Cohort follow-up (SAC) (Rosen et al., 2014) and the Prevention of Morbidity in Sickle Cell Anemia baseline investigation (POMS) (Howard et al., 2018). Inclusion and exclusion criteria have been described elsewhere (Stotesbury et al., 2022). In 2018, the TRUST sequence was added to both MRI protocols as an optional sequence for participants who tolerated the core protocol. Participants from both studies with TRUST data were eligible for inclusion.

Fetal hemoglobin (HbF) concentration, absolute reticulocyte count (ARC) and lactate dehydrogenase (LDH) were not collected as part of this study. Steady state HbF concentration, ARC and LDH were available in participants recruited from the UK National Health Service (NHS) who consented for collection of this information for research purposes and not available in the patients recruited from the community (Table 2).

**TABLE 2:** Overview of the sickle cell anemia (SCA) and healthy control (HC) subjects included in this study.

	SCA	HC
N	17	13
Age (Mean ± SD) (years)	20.1 ± 4.7	21.2 ± 3.9
Male/Female	9/8	4/9
SpO_2_ (measured/estimated)	16/1	13/0
SpO_2_ (Mean ± SD) (%)	97.5 ± 2.4	99.4 ± 0.8
Hematocrit (measured/estimated)	12/5	0/13
Hematocrit (Mean ± SD) (%)	26.3 ± 4.1	41.4 ± 3.3
Hemoglobin (Mean ± SD) (g/dl)	9.02 ± 1.47	NA
Hydroxyurea Treatment (Yes)	6 (35%)	NA
Transfusion Last 6 Months (Yes)	7 (41%)	NA
Chronic Transfusions (Yes)	3 (18%)	NA
Silent Cerebral Infarcts (Yes)	8 (47%)	2 (15%)
Previous Acute Chest Crisis (Yes) (*n* = 15)	9/15 (60%)	NA
Fetal Hemoglobin (Mean ± SD) (%) (*n* = 13)	8.75 ± 7.36	NA
Absolute Reticulocyte Count (ARC) (Mean ± SD) (cells/μl) (*n* = 8)	287 ± 105	NA
Lactate dehydrogenase (LDH) (Mean ± SD) (IU/L) (*n* = 15)	679 ± 354	NA

Ethical approval was granted by West London NHS (05/Q0408/42, 11/EM/0084, 15/LO/0347), Yorkshire NHS (15/YH/0213), and University College London (14475/001) ethics committees. Full informed consent and assent according to the Declaration of Helsinki were obtained from participants and, for children, from their parent/guardian.

### 2.2 MRI Acquisition

All participants were imaged on a 3T Siemens (Erlangen, Germany) Magnetom Prisma MRI System using a 64-channel head coil. The MRI protocol included a TRUST sequence with repetition time (TR) = 3000 ms, inversion time (TI) = 1020 ms, voxel size = 3.44 × 3.44 × 5 mm^3^, matrix size = 64 × 64 × 1, effective echo time (eTE) = 0, 40, 80, 160 ms, label slab = 100 mm and 3 averages. This was followed by a multi-parametric mapping (MPM) acquisition comprised of three fast low angle shot (FLASH) GRE sequences with respective magnetization transfer (MT), proton density (PD) and T_1_ weighting. The PD-weighted sequence parameters were: TR = 24.5 ms, TE_1_ = 2.34 ms, ΔTE = 2.34 ms, echoes = 8, flip angle = 6°, voxel size = 1 mm^3^ isotropic. T_1_-weighting was introduced by increasing the flip angle to 20° with all other parameters kept the same. MT-weighting was introduced by applying off-resonance saturation pulses prior to excitation with the number of echoes reduced to 6 to maintain consistent TR.

During the MRI examination, peripheral oxygen saturation (SpO_2_) was measured using a pulse oximeter. Blood T1 was estimated from blood hematocrit and measured SpO_2_ (Hales et al., 2016). In one SCA subject, pulse oximetry measures of SpO_2_ were not available and the mean SpO_2_ across SCA patients (97.47%) was used.

### 2.3 MRI Processing

#### 2.3.1 TRUST Processing

For each eTE, difference images were calculated between the TRUST label and control images. An initial square ROI was manually drawn covering the superior sagittal sinus (SSS). The SSS ROI was defined by selecting the four voxels with the largest signal intensity in the difference image with the shortest eTE (eTE = 0 ms). Signal intensities in the difference images were averaged over all four voxels for each eTE and estimates of the venous T_2_ (T_2b_) were estimated from a fit of these intensities as a function of eTE (Eq. 1).

Venous oxygen saturation (Y_v_) was estimated from T_2b_ using previously derived calibration models based on bovine (HbBV) and healthy (HbA) hemoglobin, as well as three models based on sickle hemoglobin; the Bush (HbS_Bush_), Li (HbS_Li_) and combined Li-Bush (HbS_Li-Bush_) models. Full details on each model are provided in the Supplementary materials.

For the 12 SCA subjects with blood samples taken, measures of hematocrit from the blood drawn closest to the MRI examination were selected. In the remaining 5 SCA subjects, hematocrit was estimated as the mean hematocrit of this cohort (26.3%).

Study ethics did not cover taking blood samples from healthy control subjects and hematocrit had to be estimated. Not all controls in this study were race-matched to the SCA participants and hematocrit estimates in white and black controls were derived from two different sources. In the white control subjects (all aged 20–30) hematocrit was estimated as 0.47 in males and 0.42 in females (Kratz et al., 2004). In the black control subjects hematocrit was estimated from a study which investigated the effect of age and sex upon blood hematcrit values in healthy black Americans (Castro et al., 1987); estimated hematocrit values ranged from 0.35 to 0.42 depending upon patient demographic.

#### 2.3.2 QSM Processing

**FIGURE 1:**
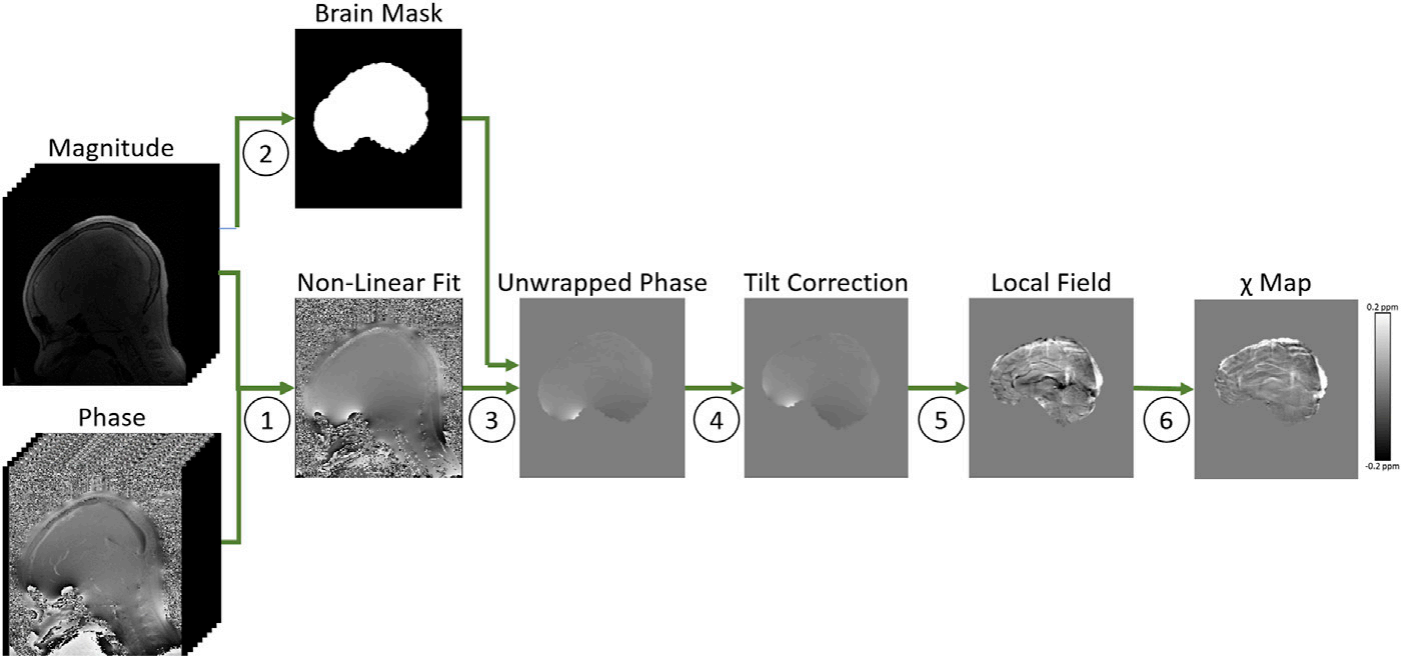
Overview of the quantitative susceptibility mapping processing pipeline in a representative healthy control, applied to the MPM T1-weighted FLASH sequence. 1. Total field maps were estimated from a non-linear fit of the complex multi-echo images (Liu et al., 2013). 2. Brain masks were estimated using FSL brain extraction tool (Smith 2002). 3. Residual wraps in the field map were removed using SEGUE phase unwrapping (Karsa and Shmueli 2018). 4. Images were realigned with the B_0_ direction (Kiersnowski et al., 2021). 5. Background field removal was performed using projection onto dipole fields (Liu et al., 2011) 6. Susceptibility maps were calculated using an iterative Tikhonov method (Karsa et al. 2020).

**FIGURE 2:**
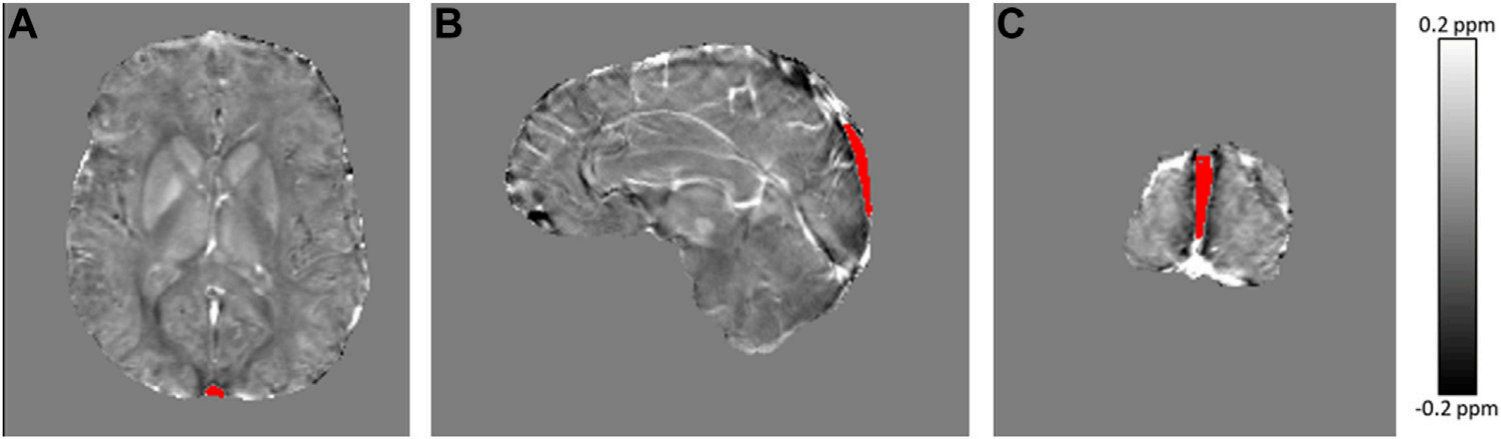
Axial **(A)**, sagittal **(B)** and coronal **(C)** view of the average susceptibility map calculated across MPM acquisitions in a representative sickle cell anemia subject, with the segmented region of interest in the superior sagittal sinus overlaid in red.

Maps of magnetic susceptibility (*χ*) were calculated from each of the three MPM FLASH acquisitions in their own native space via the following pipeline: total field maps were estimated from a non-linear fit of the complex multi-echo images (Liu et al., 2013), residual wraps in the field map were removed using SEGUE phase unwrapping (Karsa and Shmueli 2018), brain masks were estimated using FSL brain extraction tool (Smith 2002), to account for oblique acquisition images were realigned with the B_0_ direction (Kiersnowski et al., 2021) prior to background field removal via projection onto dipole fields (Liu et al., 2011) and susceptibility maps were calculated using the iterative Tikhonov method (Karsa et al., 2020) (Figure 1). To account for head movement between acquisitions, the PD- and MT-weighted images were rigidly transformed into the T_1_-w native space using NiftyReg (Modat et al., 2014). The average *χ* over the three MPM sequences was calculated to reduce noise (Murdoch et al., 2020). The mean *χ* was calculated in a ROI segmented in the SSS using a semi-automated approach in ITK-SNAP (Yushkevich et al., 2006) (Figure 2). This mean *χ* was then used to estimate Y_v_ *via* Eq. 2.

### 2.4 Statistical Analysis

Agreement between QSM and TRUST measures of Y_v_ was assessed using correlation and Bland-Altman analyses. Correlations were examined in the SCA and healthy control cohorts, in addition to the combined group. Bland-Altman analysis was carried out separately for the SCA and healthy controls groups. For each measure of Y_v_, group-wise differences between SCA and HC cohorts were also explored using Student’s t-tests.

## 3 Results

Demographic, laboratory and clinical data are presented in Table 2. Steady state HbF concentrations were available in 13/17 participants with SCA, steady state absolute reticulocyte count (ARC) in 8/17 and lactate dehydrogenase (LDH) levels in 15/17 (Table 2). Compared with our cohort study (Stotesbury et al., 2022), the proportion of patients with SCA and SCI and the rates of hydroxyurea and blood transfusion treatment were similar.

**FIGURE 3:**
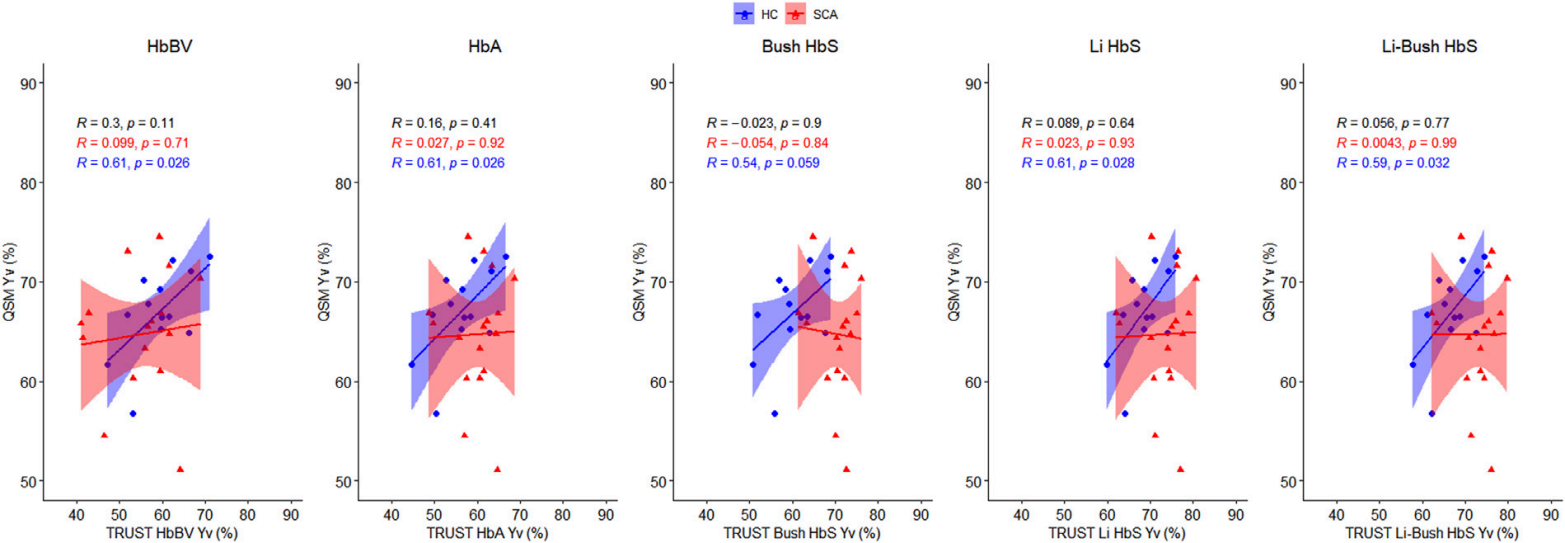
Correlation between QSM and TRUST-based measures of venous oxygen saturation (Y_v_) in sickle cell anemia (red) and healthy control subjects (blue). Pearson’s correlation coefficients (r), and the corresponding *p* values, are shown for the combined cohort (black), and for the SCA (red) and healthy control (blue) groups. Shaded areas indicate the 95% confidence intervals of the linear regression.

Correlations between the QSM and TRUST-based measures of Y_v_ in patients with SCA and healthy controls are shown in Figure 3. In healthy controls, significant correlations were observed between QSM measures of Y_v_ and each of the TRUST-based measures, except for measures based on the Bush HbS calibration (*p* = 0.059). In patients with SCA, no such correlations were observed (Figure 1). The strongest correlations in healthy controls were observed between QSM measures of Y_v_ and TRUST measures based on the HbBV (R = 0.61, *p* = 0.026) and HbA (R = 0.61, *p* = 0.026) calibration models. For the HbS calibration models in healthy controls, weaker correlations with QSM-based estimates of Y_v_ were observed. No correlations were observed between QSM- and TRUST-based measures of Y_v,_ in either the SCA or the combined group, for any of the TRUST calibration models.

**FIGURE 4:**
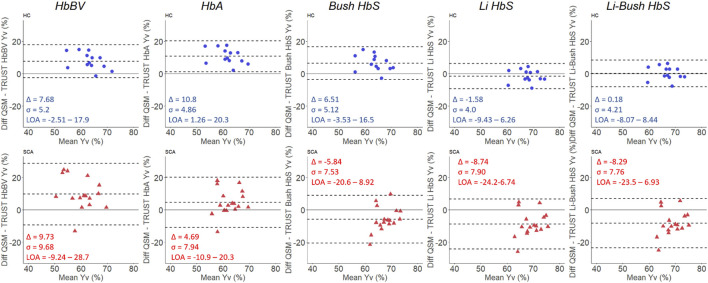
Bland-Altman plots comparing QSM and TRUST-based measures of venous oxygen saturation (Y_v_) in healthy control (blue: top) and sickle cell anemia (red: bottom) subjects. The mean bias (Δ) standard deviation of the bias (σ) and the limits of agreement (LOA) are shown for each plot.

The Bland-Altman analyses (Figure 4) showed wide variation in agreement between the QSM and TRUST-based measures of Y_v_, with agreement heavily dependent upon the calibration model used, particularly in patients with SCA. In healthy controls, QSM Y_v_ estimates were higher relative to TRUST for most of the calibration models, with mean biases (Δ) of 7.6/10.8/6.5% observed for the HbBV/HbA/HbS_Bush_ models respectively. Smaller biases of −1.6% and 0.2% were found for the HbS_Li_ and HbS_Li-Bush_ models respectively. In the SCA subjects, the direction of the mean bias varied with the calibration model. QSM Y_v_ estimates were higher relative to the HbBV and HbA calibration models (Δ = 9.7% and 4.7%) and lower relative to the three HbS models (Δ = −5.8/−8.7/−8.3%).

**FIGURE 5:**
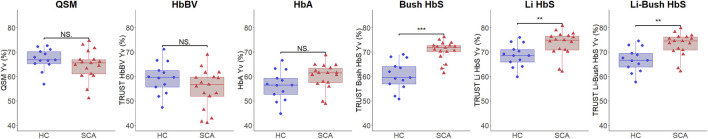
Group wise comparison of mean venous oxygen saturation (Y_v_, %, y-axis) measured in the superior sagittal sinus in sickle cell anemia (SCA) and healthy control (HC) subjects. One graph is shown for QSM of Y_v_, and TRUST-based Y_v_ measures with five different calibration models. Mean differences between groups were examined by t-test: NS: no significant difference, **p < 0.01, ***p < 0.005.

The significance and direction of group-wise differences in Y_v_ between patients with SCA and healthy controls depended on the T_2_ calibration model used (Figure 5). Although not statistically significant, both the QSM (64.7% vs. 67.0%, *p* = 0.23) and the HbBV TRUST (55.0% vs. 59.4%, *p* = 0.12) models suggested numerically decreased Y_v_ in patients with SCA relative to healthy controls. The HbA model (60.0% vs. 56.2% *p* = 0.09), and the three HbS calibration models (HbS_Bush_/HbS_Li_/HbS_Li-Bush_: 70.5% vs. 60.5%, *p* < 0.001/73.5% vs. 68.6%, *p* = 0.011/73.0% vs. 66.9%, *p* = 0.002) indicated significantly increased Y_v_ in patients with SCA relative to healthy controls.

## 4 Discussion

QSM and TRUST based measures of Y_v_ were moderately correlated in healthy controls; however poor agreement between methods was observed in patients with sickle cell anemia. Bland-Altman analyses showed the direction of biases between the QSM and TRUST-based measures of Y_v_, were dependent upon the TRUST calibration model. Group-wise Y_v_ differences between SCA patients and healthy controls were further dependent upon the method used to measure Y_v_ and were in agreement with previously reported literature.

### 4.1 Agreement Between QSM and TRUST-Based Y_v_

This study demonstrates poor agreement between measures of Y_v_ in the superior sagittal sinus derived from QSM and TRUST in patients with SCA. In patients with SCA, no significant correlations were observed between the QSM and TRUST measures, for any calibration model relating Y_v_ to the measured T_2_ of venous blood. If both TRUST and QSM were accurately estimating Y_v,_ we would expect strong correlations between the two measures.

The Bland-Altman analysis shows that QSM tended to estimate higher Y_v_ in the superior sagittal sinus relative to TRUST in the healthy control subjects for the majority of calibration models. The *χ* of venous blood is determined by the concentration of paramagnetic deoxygenated hemoglobin. Therefore, a higher estimation of Y_v_ corresponds to a lower estimation of the *χ* in the superior sagittal sinus. This lower estimation may have been caused by inaccuracies in QSM in the SSS due to its location at the periphery of the brain mask. Any field perturbations outside of the brain, caused by the deoxygenated hemoglobin in the SSS, cannot be sampled, which will reduce the accuracy of *χ* and Y_v_ estimates in the vein. One of two published QSM studies of Y_v_ in SCA (Shen et al., 2019) considered *χ* values in the straight sinus and reported a mean Y_v_ of 69.3% vs. 73.9% in SCA vs. HC compared to 64.7% vs. 67.0% in the SSS in our study. Despite the straight sinus being far away from the edge of the brain mask, the estimates of Y_v_ in the straight sinus were even higher than in the SSS in our study. This may have been caused by partial volume effects in this venous structure, which is smaller than the SSS and more challenging to accurately segment, or could have been due to the use of L1 regularization in the *χ* map calculation which is known to underestimate venous *χ* (Berg et al., 2021). The choice of susceptibility calculation method has been shown to have a significant effect on estimates of Y_v,_ with Tikhonov-based regularization, as used in this study, shown to be the most accurate method for venous susceptibility measurement in a recent study (Berg et al., 2021).

In the SCA subjects, the direction of the bias between QSM and TRUST methods was dependent upon the calibration model employed. QSM Y_v_ measures were increased relative to the HbBV and HbA models and reduced relative to the three HbS models. Examining the Bland-Altman analysis, only the limits of agreement from the QSM-TRUST HbA comparison in healthy controls excluded zero and demonstrated a systematic bias.

### 4.2 Y_v_ in SCA vs. Healthy Controls

**FIGURE 6:**
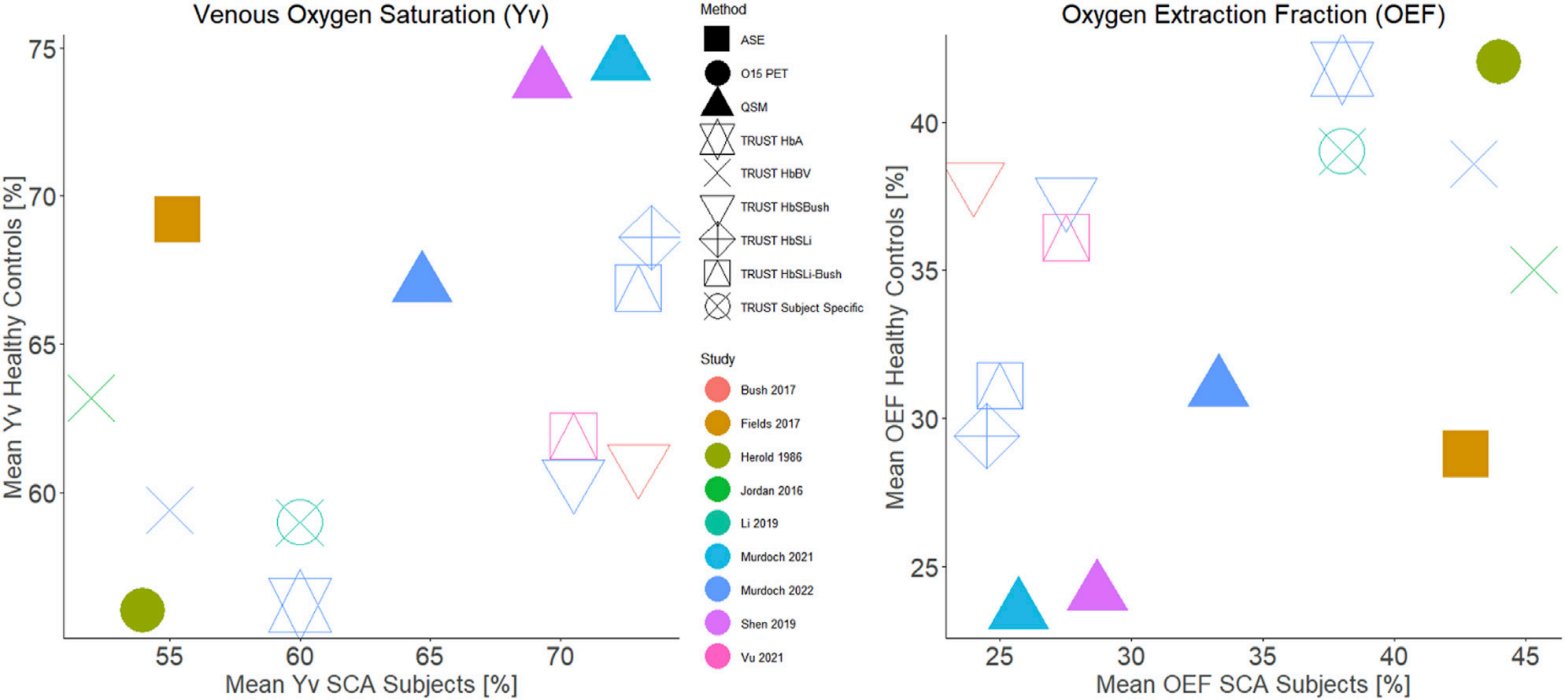
Comparison of group mean venous oxygen saturation **(left)** and oxygen extraction fraction **(right)** in sickle cell anemia and healthy controls cohorts reported in previous literature. The points are colour coded by study and have symbols corresponding to the measurement technique used. For studies which did not report both the oxygen extraction fraction and venous oxygen saturation, the missing values were calculated assuming an arterial oxygenation of 98%.

The results of the group-wise comparisons of Y_v_ in patients with SCA versus healthy controls largely agree with the results of previous studies investigating the effect of SCA on Y_v,_ and a recent comparison of the models in a pediatric population (Lin et al., 2022)_._ An overview of these previous studies investigating Y_v_ in SCA is shown in Table 1, with results visualized in Figure 6. In the present study, the QSM results suggested numerically decreased Y_v_ in SCA relative to healthy controls. These results are in agreement with two prior studies using QSM to estimate Y_v_ in SCA (Shen et al., 2019; Murdoch et al., 2021). TRUST with the HbBV calibration model similarly suggested numerically decreased Y_v_ in SCA versus healthy controls; in agreement with the prior TRUST study in SCA that employed this model (Jordan et al., 2016). A decrease in Y_v_ in patients with SCA relative to healthy controls was also reported in a whole brain study that applied an asymmetric spin-echo based MRI-method and found that whole brain OEF was increased in SCA relative to HC (42.7% vs. 28.8%) (Fields et al., 2018), which is equivalent to a decrease in Y_v_ in SCA. However, in the present study, neither the QSM or TRUST HbBV Y_v_ differences between patients with SCA and HC reached significance.

Conversely, TRUST with each of the HbS calibration models found Y_v_ to be significantly increased in patients with SCA relative to healthy controls. TRUST with the HbA model also suggested elevated Y_v_ in SCA but the difference did not reach significance. Our TRUST results using HbS calibration models are in agreement with the results of two TRUST studies in larger cohorts which applied the HbS_Bush_ and HbS_Li-Bush_ calibration models respectively (Bush et al., 2018; Vu et al., 2021).

Oxygen metabolism in the brain is measured by the cerebral metabolic rate of oxygen (CMRO_2_) which is given by

$$CMRO_{2}=C_{a}\cdot OEF\cdot CBF$$ (3)

where C_a_ is the arterial oxygen content, the product of $C_{a}$ and OEF is the arteriovenous difference in oxygen saturation, and CBF is the cerebral blood flow.

CMRO_2_ must be maintained to meet the oxygen demands of the brain, and ischemic stroke occurs when insufficient oxygen is delivered to tissue. In SCA, $C_{a}$ is diminished as result of hemolysis and the corresponding reduction in blood hematocrit. It has been shown that CBF increases in SCA to compensate for the reduced arterial content (Bush et al., 2016). It has been hypothesized that OEF might increase in SCA to overcome the reduced C_a_ caused by anemia and in some cases oxygen desaturation. The studies reporting a decreased OEF in SCA have attributed this change to arteriovenous shunting (Stotesbury et al., 2019). If OEF is decreased in SCA, corresponding to increased Y_v_, any CBF increase must be sufficiently large to compensate for the decreased C_a_ and OEF and ensure sufficient oxygen is supplied to tissue. There is little evidence that CMRO2 is diminished in SCA as the only published O^15^ PET study comparing patients with SCA and healthy controls reported no significant CMRO_2_ differences between the two cohorts (Herold et al., 1986).

### 4.3 Validation of QSM and TRUST-Based Measures of Y_v_

One limitation of our study is the lack of a gold-standard measure of Y_v,_ such as O^15^ PET or jugular vein catheterization, which precluded investigation of the relative accuracy of the QSM and TRUST methods with each of the calibration models, beyond comparison with previously reported values. The Herold et al. PET study found no significant OEF differences between SCA and healthy control groups (SCA: 44% vs. HC: 42%). Y_v_ measures from QSM and TRUST with each of the calibration models can be compared with O^15^ PET estimates of Y_v_: OEF = 44% corresponds to Y_v_ = 54% for an assumed arterial oxygen saturation of 98%. In patients with SCA, the TRUST HbBV model provided the closest estimate to the reported PET value (55%) whilst the TRUST method using the Li HbS and Li-Bush HbS calibration models (Y_v_ = 73.5% and 73.0% respectively), had the largest difference and may plausibly overestimate Y_v_ in SCA (Figure 6). However, it must be noted that the Herold study was conducted in a small population, and additional confounding factors will affect Y_v_ measures, including patient treatment.

In SCA patients, neither TRUST nor QSM measures of Y_v_ have been validated against gold standard measures. In other populations, QSM-derived OEF has also been compared with gold-standard O^15^ PET measures of OEF. Kudo et al. compared whole brain OEF measured from QSM using an approach reported by Zaitsu et al. (2011) to the gold-standard O^15^ PET OEF Kudo et al. (2016). The whole brain OEF maps were estimated from QSM by thresholding the *χ* maps to extract a venous map, and Y_v_ was estimated within each vein from the difference between the mean *χ* in each venous voxel relative to the *χ* in the surrounding brain parenchyma. OEF was estimated from 1-Y_v_ and a sliding window method was applied within slices to generate venous OEF maps. Significant correlations were observed between QSM and PET OEF measures at a hemispheric level, and QSM-OEF was sensitive to increased hemispherical OEF in patients with chronic unilateral internal carotid or middle cerebral artery stenosis or occlusion.

### 4.4 Limitations of QSM and TRUST Estimation of Y_v_ in SCA

TRUST can currently only provide an estimate of global Y_v_ from just a few voxels whereas QSM can provide Y_v_ estimates throughout the venous vasculature. The relatively large dimensions of the TRUST voxels (∼59 mm^3^) relative to the QSM voxels (1 mm^3^) limited the number of voxels used to estimate venous T_2_ to 4, whilst the mean number of voxels used to estimate the mean *χ* was orders of magnitude greater (mean ± standard deviation = 1225 ± 793). QSM can be affected by partial volume effects, with the inclusion of non-blood tissue within the ROI likely to result in reduction of the apparent venous *χ* and overestimation of Y_v_. However, the large size of the superior sagittal sinus compared to the QSM voxel size minimizes the risk of partial volume effects (McFadden et al., 2021). In contrast to QSM, the image subtraction performed in TRUST removes signal from static tissue, ensuring that only venous blood contributes to the measured signal.

The TRUST signal model outlined in Eq. 1 shows that the TRUST signal differences are dependent upon both T_1_ and T_2_ relaxation times. The majority of previous studies have estimated the venous blood T_1_ from measured hematocrit levels but T_1_ is also dependent on the venous blood oxygenation (Hales et al., 2016). In our study, venous blood T_1_ was estimated using a model developed by Hales et al. (Hales et al., 2016) based on the estimated hematocrit and arterial oxygen saturation measured using pulse oximetry. Pulse oximetry provides an estimate of arterial oxygenation but it is unlikely to accurately represent the venous oxygen saturation, adding another potential confound into TRUST oxygenation measurements.

An alternative method for estimating blood T_1_ is to use MRI multi-parametric mapping (MPM), a quantitative imaging method which provides high resolution maps of parameters including T_1_ (Lutti et al., 2013). This method is likely more accurate than using the model based on hematocrit and peripheral arterial oxygenation. Here, we used MPM T_1_ maps to calculate the mean T_1_ within the SSS ROI (used to estimate venous χ), to provide an alternative measure of blood T_1_. Despite differences between the MPM-measured and Hales model-estimated blood T_1_ values, these had a negligible effect on the T_2b_ values estimated by fitting Eq. 1 (Supplementary Materials). This suggests that using estimated blood T_1_ values was not a substantial source of error/inaccuracy in the TRUST results in this study.

The study by Li et al. suggested that individual TRUST T2 calibration models may be required for each SCA patient as blood T2 is dependent upon factors beyond Hct and Yv (Li et al., 2020). Red blood cell shape and the concentration of fetal hemoglobin in the blood were suggested as potential confounds to the T2 calibration model. Fetal haemoglobin (HbF) differs from adult haemoglobin (HbA) in the globular protein subunits: HbA is comprised of two alpha and two beta subunits, whilst HbF consists of two alpha and two gamma subunits. As the magnetic susceptibility of hemoglobin is determined by oxygenation dependent differences in the electron configuration of the heme iron centres, it is probable that the magnetic susceptibility of deoxygenated HbA and HbF are comparable, but this will require validation.

Hydroxyurea and blood transfusions are known to affect the relative concentrations of HbF and HbA and, given that HbF has a higher oxygen affinity than HbA, these therapies may, therefore, influence venous oxygen saturation. For the 13 patients with sickle cell anemia where steady state fetal hemoglobin levels were available, only 1 patient receiving hydroxyurea had unusually elevated HbF levels (>20%). Therefore, elevated HbF levels in the SCA cohort are unlikely to have had a confounding effect on the groupwise comparison results shown in Figure 5.

Future validation of QSM measures of Yv in patients with SCA would be strengthened by measuring the hemoglobin composition to investigate any potential confounding effects of the proportion of HbF/HbA/HbS.

Blood rheology has been shown to be modified in patients with sickle cell anemia (Connes et al., 2016). QSM and TRUST models are dependent upon blood hematocrit levels, a key determinant of blood viscosity. However, the possible effects of blood rheology on venous T2, flow and oxygenation in SCA or the potential effects of increased fibrinogen levels on blood susceptibility have not been investigated and could be explored in future work.

Accurate MRI measures of venous oxygen saturation in patients with sickle cell anemia would provide a useful biomarker when evaluating the efficacy of novel treatments. Of particular interest would be hemoglobin oxygen modifying therapies, for example Voxelotor (Oxbryta), which aims to reduce sickling by stabilizing HbS in an oxygenated state (Herity et al., 2021). It would be expected that prevention of HbS sickling would increase arterial oxygen capacity and, therefore, venous oxygen saturation, which could be quantified using the MRI measures compared in this study.

This study is limited by the relatively low numbers of patients with SCA and healthy controls, as well as by the use of estimated hematocrit values for 5/17 SCA subjects and all the healthy control subjects. QSM measures of Y_v_ were restricted to the SSS for the purpose of comparison with the TRUST results, which are limited to the SSS due to the low resolution, coverage and SNR of the TRUST MRI acquisition. QSM can provide local measures of Y_v_ in any venous structures which can be segmented (McFadden et al., 2021). We would expect localized changes in Y_v_ in SCA, as evidenced by the high incidence of ischemic stroke in the internal carotid and middle cerebral artery territories (Adams 1995; Pavlakis and Levinson 2009). Any localized changes in Y_v_ in SCA may only have a small effect in the SSS as many veins drain into the SSS, and future work should consider applying QSM to measure Y_v_ beyond the major draining veins.

## 5 Conclusion

This study compares QSM and TRUST measures of venous oxygenation (Y_v_) in patients with sickle cell anemia. QSM and TRUST-based Y_v_ in the superior sagittal sinus were significantly correlated in healthy controls using all but one TRUST calibration model (HbS_Bush_). However, in patients with sickle cell anemia, we observed disagreement between QSM and TRUST measures of venous oxygen saturation in the superior sagittal sinus. QSM measures of Y_v_ suggested decreased Y_v_ in SCA relative to healthy controls in agreement with TRUST measures of Y_v_ using a bovine hemoglobin calibration model and previous studies using asymmetric spin-echo methods. This is opposite to the increased Y_v_ in SCA relative to healthy controls found here and in previous studies using TRUST methods based on sickle hemoglobin calibration models. Further validation of all MRI measures of Y_v_, relative to ground truth measures such as O^15^ PET and jugular vein catheterization, is required in SCA before QSM or TRUST methods can be considered for neurological risk prediction in clinical practice.

## Data Availability

Full anonymized data will be shared on request from qualified investigators.
