# Supplementary material for: A Comparison of MRI Quantitative Susceptibility Mapping and TRUST-Based Measures of Brain Venous Oxygen Saturation in Sickle Cell Anaemia
Source: Front Physiol. 2022 Aug 29;13:913443. doi: 10.3389/fphys.2022.913443 (PMC9465016; doi:10.3389/fphys.2022.913443)
Supplement: Supplementary file 1 [file Table1.DOCX]

Supplementary Material

# Supplementary Data

## TRUST T2 Calibration Models

### Bovine Haemoglobin (HbBV)

TRUST was first developed with a calibration model calculated from bovine haemoglobin (HbBV). The T2 calibration model was based on the two-compartment exchange model developed by Wright et al. and Golay et al. The relationship between the T2 of venous blood (T2_b_) and the venous oxygen saturation (Y_v_) is given by:

$$\frac{1}{T2_{b}}=A+B\cdot\left( 1-Y_{v} \right)+C\cdot\left( 1-Y_{v} \right)^{2}$$

Where A, B and C are dependent on blood haematocrit (Hct)

$$A=a_{1}+a_{2}\cdot Hct+a_{3}\cdot Hct^{2}$$

$$B=b_{1}\cdot Hct+b_{2}\cdot Hct^{2}$$

$$C=c_{1}\cdot Hct\cdot\left( 1-Hct \right)$$

The values of A, B, and C are dependent upon the inter echo spacing of the Carr-Purcell-Meiboom-Gill T2 preparation pulse (τ_CPMG_). For τ_CPMG_ = 10ms, a_1_ = -13.5, a2 = 80.2, a3 = -75.9, b1 = -0.5, b2 = 3.4, and c1 = 247.4 [all s^-1^].

### Healthy Haemoglobin (HbA)

The empirical model derived from healthy haemoglobin (HbA) extended the HbBV model to incorporate the relationship between haematocrit levels and the T2 of fully oxygenated blood. The updated model is given by:

$$\frac{1}{T2_{b}}=A_{1}\cdot Hct\cdot\left( 1-Y_{v} \right)^{2}+A_{2}\cdot\left( 1-Y_{v} \right)^{2}+A_{3}\cdot Hct+A_{4}$$

For τ_CPMG_ = 10ms, A_1_ = 77.5, A_2_ = 27.8, A_3_ = 6.95, A_4_ = 2.34.

### Bush Sickle Haemoglobin (HbS_Bush_)

The first calibration study performed on sickle haemoglobin (HbS) found that neither the HbBV or HbA models adequately fit the data from sickle blood. Therefore, the following haematocrit independent model was proposed instead:

$$\frac{1}{T2_{b}}=A\cdot\left( 1-Y_{v} \right)^{2}\cdot B$$

For τ_CPMG_ = 10ms, A = 70.0, B = 5.75.

### Li Sickle Haemoglobin (HbS_Li_)

A study by Li et al, evaluated the use of individual T2 calibrations to measure Y_v_ in SCA subjects. A group wise calibration model was retrospectively calculated for the 12 SCA subjects given by:

$$\frac{1}{T2_{b}}=278\cdot Hct\cdot\left( 1-Y_{v} \right)^{2}-5\cdot\left( 1-Y_{v} \right)^{2}-8.4\cdot Hct+9.3$$

### Li-Bush Sickle Haemoglobin (HbS_Li-Bush_)

Participants from the Bush et al and Li et al. studies were pooled to develop a more robust calibration model derived from the increased patient cohort. Separate models were suggested for transfused and non-transfused patients. The combined model suggested for non-transfused patients is as follows:

$$\frac{1}{T2_{b}}=196.8\cdot Hct\cdot\left( 1-Y_{v} \right)^{2}+16.7\cdot\left( 1-Y_{v} \right)^{2}-6.6\cdot Hct+8.6$$

# Supplementary Figures and Tables

For more information on Supplementary Material and for details on the different file types accepted, please see [here](http://home.frontiersin.org/about/author-guidelines#SupplementaryMaterial). Figures, tables, and images will be published under a Creative Commons CC-BY licence and permission must be obtained for use of copyrighted material from other sources (including re-published/adapted/modified/partial figures and images from the internet). It is the responsibility of the authors to acquire the licenses, to follow any citation instructions requested by third-party rights holders, and cover any supplementary charges.

## Supplementary Figures

**
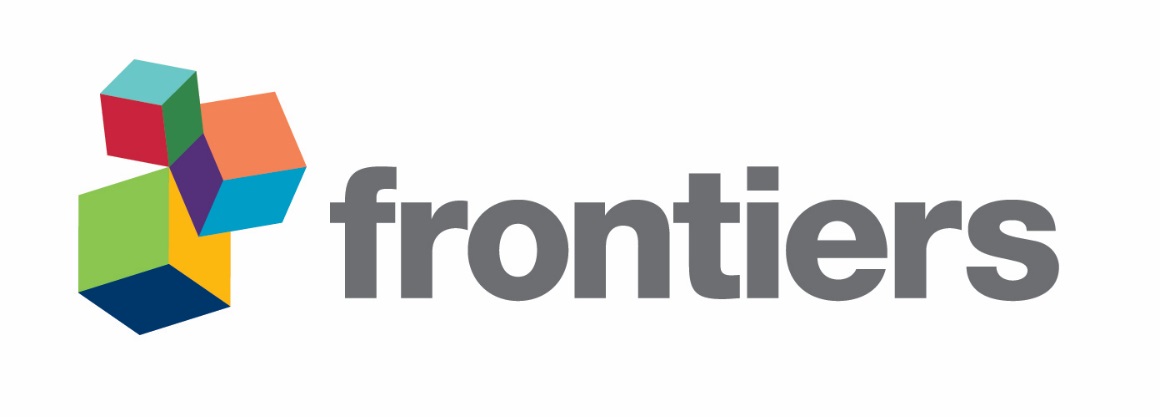
**

**Supplementary Figure 1.** The figure legends are required to have the same font as the main text, 12 point normal Times New Roman, single spaced. Please use a single paragraph for each legend and prepare the figures keeping in mind the PDF layout.

**
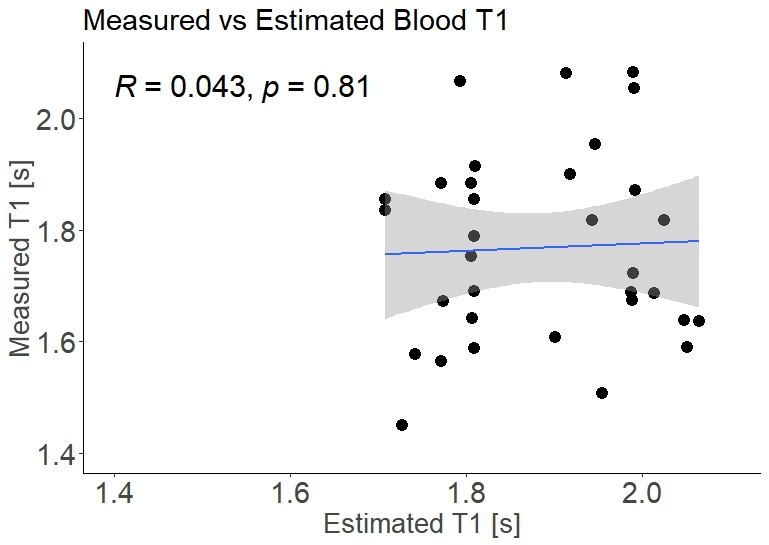
**

Supplementary Figure 1: Comparison of blood T1 estimated from blood haematocrit and peripheral oxygenation (x-axis) and blood T1 measured using multi-parametric mapping in a region of interest in the superior sagittal sinus.

**
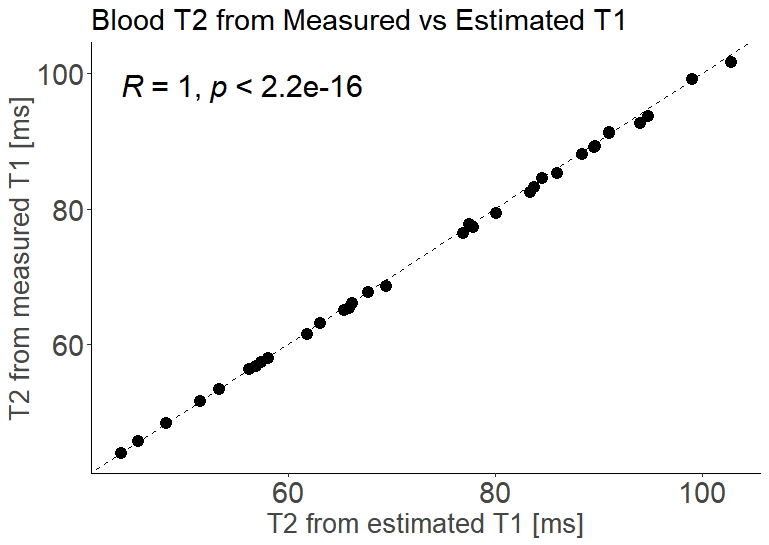
**

Supplementary Figure 2: Comparison of Blood T2 calculated from an exponential fit of the TRUST signal, using the estimated (x-axis) and measured (y-axis) blood T1 values. The dashed line represents the line of unity between the two measures. The strong correlation between T2 values calculated using the estimated and measured T1 values suggests that estimating blood T1 does not introduce a significant error into the blood T2 measures.
